# Fully Bayesian tests of neutrality using genealogical summary statistics

**DOI:** 10.1186/1471-2156-9-68

**Published:** 2008-10-31

**Authors:** Alexei J Drummond, Marc A Suchard

**Affiliations:** 1 Bioinformatics Institute, University of Auckland, Private Bag 92019, Auckland, New Zealand; 2 Department of Computer Science, University of Auckland, Private Bag 92019, Auckland, New Zealand; 3 Departments of Biomathematics and Human Genetics, David Geffen School of Medicine at UCLA, Los Angeles, California, USA; 4 Department of Biostatistics, UCLA School of Public Health, Los Angeles, California, USA

## Abstract

**Background:**

Many data summary statistics have been developed to detect departures from neutral expectations of evolutionary models. However questions about the neutrality of the evolution of genetic loci within natural populations remain difficult to assess. One critical cause of this difficulty is that most methods for testing neutrality make simplifying assumptions simultaneously about the mutational model and the population size model. Consequentially, rejecting the null hypothesis of neutrality under these methods could result from violations of either or both assumptions, making interpretation troublesome.

**Results:**

Here we harness posterior predictive simulation to exploit summary statistics of both the data and model parameters to test the goodness-of-fit of standard models of evolution. We apply the method to test the selective neutrality of molecular evolution in non-recombining gene genealogies and we demonstrate the utility of our method on four real data sets, identifying significant departures of neutrality in human influenza A virus, even after controlling for variation in population size.

**Conclusion:**

Importantly, by employing a full model-based Bayesian analysis, our method separates the effects of demography from the effects of selection. The method also allows multiple summary statistics to be used in concert, thus potentially increasing sensitivity. Furthermore, our method remains useful in situations where analytical expectations and variances of summary statistics are not available. This aspect has great potential for the analysis of temporally spaced data, an expanding area previously ignored for limited availability of theory and methods.

## Background

The field of population genetics has a long history in the development of tests of selective neutrality. This is both because of the difficulty of developing a tractable alternative to the neutral theory and because of the ongoing debate about how well the neutral theory can explain real data. Although a number of important steps have been made to develop powerful tests of neutrality [1-3] there are evident problems with many currently available tests. For example many of the tests, such as Tajima's *D *(*D*_*T*_) and Fu and Li's *D *(*D*_*F*_) have difficulty in accurately discriminating between selection and changes in population size.

In fact, most available tests of neutrality can only test constant population size neutrality against alternatives that include both population growth and selection. Furthermore, most tests require accurate knowledge of the number of mutations that have occurred or the branch lengths in the gene tree, and do not adequately take into account the uncertainty in these quantities (i.e. most tests implicitly assume an infinite-sites model of evolution). Finally, tree-based summary statistics are often based on one estimate of the genealogy, despite the fact that the true genealogy and branch lengths are seldom known.

Broadly speaking, on the basis of the sequence information used, statistics for testing neutrality can be placed into three classes:

1. statistics that use the mutation (segregating site) frequency spectrum [1,2,4,5],

2. statistics that use the haplotype distribution [3,6,7] and

3. statistics that use the pair-wise distance (mismatch) distribution [8,9].

A recent comprehensive survey of the power of these different classes of tests for detecting population expansion found that classes 1 and 2 were generally more powerful than the best class 3 statistics [10]. Some of the best-known test statistics come from class 1 and essentially work by comparing aspects of the mutation frequency spectrum with neutral expectations. This class of test statistics include *D*_*T *_[1], *D*_*F *_[2] and the *H *statistic [5]. In the simplest case, these statistics can be used to measure deviations from the null hypothesis of constant population size, random mating and no recombination. For example *D*_*F *_measures the normalized difference between the number of mutations on the external branches and the total number of mutations in the genealogy. Under the null hypothesis of neutral evolution the expectation of *D*_*F *_is zero, and a significant departure from zero signifies selection (balancing, directional, negative), recombination or changes in population size. The last of these alternatives is problematic because exponential growth is expected to give results similar to directional or purifying selection. For this reason it would seem desirable to develop a method that directly accounts for alternative demographic models of population size through time. In this context, several studies have combined the use of summary statistics and demographic models [11-15].

Apart from biasing the mutation frequency distribution, selection may also affect the shape of the gene tree [16]. Although few attempts have been made to use this expectation in a rigorous test of neutrality (c.f. [17]), a number of branching models and summary statistics measuring tree imbalance exist in the literature of speciation models [18-21]. A method that could use information both from the mutation frequency spectrum and from the shape of the gene tree may be more powerful than either used individually.

If all sequences comprising a gene tree are sampled from the same time point (as is required by most tests of neutrality) then there is very little power to distinguish between selection and exponential growth. However if sequence data is available from different times, during which measurable evolution has taken place, as in RNA viruses and ancient mitochondrial DNA (mtDNA) data [22,23] then the power to distinguish between these two alternatives is potentially much greater. Unfortunately, the expectations and variances of crucial quantities (such as tree length) are not yet available for serially sampled data, so this potential power has not been tapped.

Apart from analyses of intra-population sequence variation, evidence for non-neutrality can also be detected by comparing within- and between-species sequence variation [24]. For example, it has been widely observed that in some species there is an excess number of polymorphic non-synonymous sites segregating within the species relative to the number of non-synonymous sites with fixed differences between closely related species [19,25]. This effect is consistent with the conclusion that a substantial fraction of non-synonymous mutations are slightly deleterious mutations (SDMs) that often persist as polymorphisms within populations for some time but have a low probability of eventual fixation [26]. However this pattern is not universal. In fact, at least in *Drosophila *the pattern appears to be the reverse [27], possibly implying a prominent role for recurring positive selection [28]. Regardless of the direction of non-neutral evolution this test may suggest, it has been shown that, as with summary statistics of the mutation frequency spectrum, the accuracy of these methods is compromised by the effects of unrecognized historical demographic change [29]. Both within-species and between-species methods rely on the fact that SDMs become increasingly rare relative to neutral mutations at higher frequencies. For example, within a panmictic population, the distribution of SDMs is expected to predominate near the tips of a population genealogy [30], so that SDMs are on average younger than neutral mutations [25]. Thus the older branches (and associated mutations) within a population will tend to consist of relatively fewer SDMs (as purifying selection has had longer to act).

Although a number of researchers have observed non-neutral behaviour of non-synonymous polymorphism in protein-coding regions, few have considered the effect of SDMs on linked genetic variation in non-coding regions. This is particularly pertinent to the study of the control region of mitochondrial genes, which is extensively used for within-population genetic sampling of animal mtDNA [31]. The action of Hill-Robertson interference is expected to exacerbate the persistence of SDMs in populations [32], because it reduces the efficiency of purifying selection. Even moderately deleterious mutations, which would otherwise be removed by selection very quickly, can persist in the population if there is substantial genetic linkage between sites [30]. Therefore, in non-recombining genetic elements such as the mitochondrial genome and the genomes of negatively stranded RNA viruses, mutations that are themselves selectively neutral will nevertheless tend to share the fate of linked deleterious mutations.

In this paper we extend an existing Bayesian method originally applied to investigating non-neutrality in HIV evolution [33], that can be used to test for selective neutrality in both coding and non-coding genetic regions sampled from within a single population. The method assumes no knowledge of ancestral mutation frequencies and takes into account the confounding effects of demographic history. We demonstrate the utility of this method on four examples comprised of one non-coding data set and three coding data sets. This method assumes a single genealogy describes the evolutionary history of the sequences under study, but makes no assumptions about ancestral mutation frequencies and takes into account the confounding effects of demographic history. We demonstrate the utility of this method on four non-recombinant examples comprised of one non-coding data set and three coding data sets.

## Results and discussion

We employed a suite of summary statistics to test the assumption of neutrality on four example data sets. Because selection is expected to change both the distribution of mutations on the tree and the shape of the sample genealogy [30], statistics that measure both of these departures were included in the analysis.

### Summary statistics

Fu and Li [2] compared two estimates of population parameter *θ *that can be derived for a sample of *n *sequences:

1. the total number of singleton polymorphisms and

2. the total number of segregating sites divided by $a_{n}=\sum_{k=1}^{n-1}k^{-1}$.

Under neutrality the difference between these two measures is expected to be zero, and the variance in the difference can be calculated. The resulting normalized test statistic *D*_*F *_assumes an infinite sites model of mutation, because it equates mutations with branch lengths in the underlying coalescent tree and does not therefore account for the possibility of multiple mutations at a single site. To avoid this assumption we employ a genealogy-based version of *D*_*F*_, which compares the length of terminal branches to the total length of the coalescent genealogy (we term this the *genealogical D*_*F*_). In addition to the genealogical *D*_*F*_, two other measures of branch length distribution (age of most recent common ancestor, and total tree length; see Table 1) and three measures of tree imbalance *B*_1_, *I*_*c *_and *C*_*n *_were also employed.

**Table 1:** Summary statistics used in test of neutrality

Summary Statistic	Reference	Description
*T*	-	The total length of all branches of the tree.
*t*_MRCA_	-	The difference in age between the most recent common ancestor and the most modern individual.
*D*_*F*_	[2]	A classic summary statistic for testing neutrality. Normalized difference between external branch lengths and total tree length.
*B*_1_	[34]	A measure of tree-imbalance.
*C*_*n*_	[20]	The number of internal nodes with exactly two terminal children(the number of cherries).
*I*_*c*_	[18]	A measure of tree-imbalance. Ranges is [0,1]. Larger numbers signify more imbalanced trees.

The *B*_1 _statistic is the maximum number of nodes between an internal node and the tips of the tree, summed over all internal nodes and excluding the root [34]. Higher values of *B*_1 _are expected with increasing symmetry of the phylogeny. Colless's tree imbalance index *I*_*c *_considers each internal node of a bifurcating tree and partitions the number of terminal sequences that descend from it into two groups, *r *and *s*, where *r *≥ *s*. Symmetry is measured based on the difference between *r *and *s*, summed over all internal nodes [18]. The measure increases from 0 for a perfectly symmetrical tree, to 1 if the tree is completely asymmetric. The final tree-asymmetry measure, Cherry count *C*_*n*_, is simply the number of pairs of sequences joined by their most recent common ancestor [20]. More symmetrical trees are identified by higher values of *C*_*n*_. All six summary statistics used are listed in Table 1.

### Data analysis

#### Brown bear mitochondrial DNA

An alignment of non-coding mitochondrial DNA from the d-loop of brown bears *Ursus ursus *was compiled as an example of non-coding molecular sequence data that is assumed to be evolving neutrally. The data set comprised 30 previously published ancient DNA sequences [35], along with 44 modern brown bear sequences obtained from GenBank. The software BEAST [36] was used to conduct Bayesian MCMC analysis on the full data set (n = 74), yielding estimates of evolutionary rate, population size and ancestral genealogy (Table 2). The substitution model chosen allowed for different rates of transitions and transversions [37] as well as Γ-distributed rate heterogeneity among sites [38]. Both constant-size and exponential-growth models of demography were investigated. To test if the assumption of neutrality was warranted, posterior and posterior predictive values were calculated for each of the summary statistics in Table 1, along with their corresponding multivariate posterior predictive *p*-value. A Bayes factor computed via importance sampling [39,40] was used as a model choice criterion to compare the relative marginal likelihoods of the two models, resulting in rejection of the exponential growth model in favour of a constant-size population. However under both models, differences between the posterior and posterior predictive values did not suggest any significant departures from neutrality in any of the six summary statistics investigated. The multivariate *p*-values for constant and exponential growth were 0.219 and 0.284 respectively. This result suggests, at least in terms of tree asymmetry and branch length distribution, that selective neutrality cannot be rejected for the d-loop of brown bears.

**Table 2:** Bayesian parameter estimates

Data Set	Demographic Model	log *P*(*D*|*M*)^1^	*N*_*e*_*τ *(years)	*r*^2^	*μ*^3^	*α*^4^	*κ*	*t*_*MRCA *_(years)
Brown bear (d-loop)	Constant*	-2200	113,800	-	5.68 × 10^-7^	0.243	41.8	153,500
	Exp. growth	-2198	127,000	5.45 × 10^-6^	5.95 × 10^-7^	0.243	41.7	145,100
HRSV (g gene)	Constant*	-6068	36.3	-	0.00242	0.900	12.4	56.1
	Exp. growth	-6070	53.0	0.0263	0.00239	0.900	12.4	55.8
Dengue-4 (E gene)	Constant	-3960	11.2	-	0.000976	0.167	17.3	19.7
	Exp. growth*	-3952	38.9	0.134	0.00096	0.167	17.2	19.0
Influenza A (HA)	Constant*	-4386	4.3	-	0.00503	0.332	5.49	19.0
	Exp. growth	-4383	7.25	0.0681	0.00506	0.332	5.5	18.9

Posterior parameter estimates from the MCMC analyses. The effective population size is reported only as a product with generation time (*N*_*e*_*τ*) and the compound parameter has unit of years for virus data sets and radiocarbon years for the brown bear data set. Posterior means are reported for all model parameters. For each data set, the demographic model chosen by a Bayes factor is marked (*). ^1^marginal likelihood, ^2^exponential growth rate, ^3^substitution rate, ^4^shape parameter of the Γ-distribution.

#### RNA virus data sets

Three RNA virus data sets were also analyzed under the same model conditions as described above. The first was a multiple sequence alignment (*n *= 129) of the g gene (*L *= 629 bp) of human respiratory syncytial virus (HRSV) spanning 46 years from 1956 to 2002 [41]. This virus was used as an example of a coding gene of an RNA virus that exhibits only a weak signal of non-neutrality in terms of its tree shape. The estimates of mutation rate and population size are shown in Table 2. A constant population size was preferred over exponential growth using a Bayes factor. The multivariate posterior predictive *p*-values did not reject neutrality (*p *= 0.33). We followed up with a series of univariate analyses using the individual summary statistics. The tree length *T*, age of the root *t*_MRCA _and *D*_*F *_statistics are all close to significance under the assumption of constant population size, as shown in Table 3, while the remaining univariate statistics are less suggestive. Therefore, there is only marginal evidence for low levels of non-neutrality in the tree shape of HRSV.

**Table 3:** Predictive Probabilities

Data Set	Demographic Model	*T*	*t*_MRCA_	*D*_*F*_	*I*_*c*_	*C*_*n*_	*B*_1_	MV
Brown bear (d-loop)	Constant	0.205	0.164	0.128	0.307	0.844	0.900	0.219
	Exponential growth	0.382	0.374	0.209	0.305	0.832	0.886	0.284
HRSV (g gene)	Constant	0.045	0.034	0.044	0.835	0.851	0.865	0.330
	Exponential growth	0.294	0.335	0.121	0.805	0.845	0.857	0.463
Dengue-4 (E gene)	Constant	0.036	0.004*	0.001*	0.434	0.401	0.581	0.027*
	Exponential growth	0.219	0.170	0.013*	0.449	0.349	0.498	0.128
Human influenza A (HA)	Constant	0.040	0.101	<0.001*	0.951	0.392	0.393	0.024*
	Exponential growth	0.085	0.381	0.001*	0.916	0.427	0.438	0.018*

Univariate and multivariate posterior predictive *p*-values for summary statistics on each of the example data sets. Significant departures (univariate: *p*_*B *_<*α*/2 or *p*_*B *_> 1 - *α*/2; multivariate: *p*_*B *_<*α *for *α *= 0.05) from neutrality are marked (*). Significant departures on the best fitting model for each data set are in bold.

**Figure 1:**
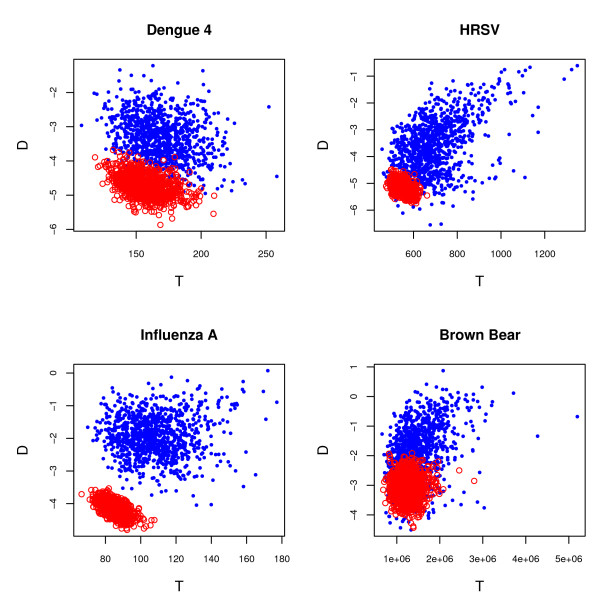
**Posterior and predictive distributions of tree length *T *and *D*_*F*_**. Posterior and predictive distributions of tree length *T *and *D*_*F *_for all four data sets. The dengue-4 data is from an analysis assuming exponential growth, while the other three analyses assumed a constant population size. Human influenza A virus shows the largest departure from neutrality, with the posterior distribution completely disjoint from the predictive distribution.

**Figure 2:**
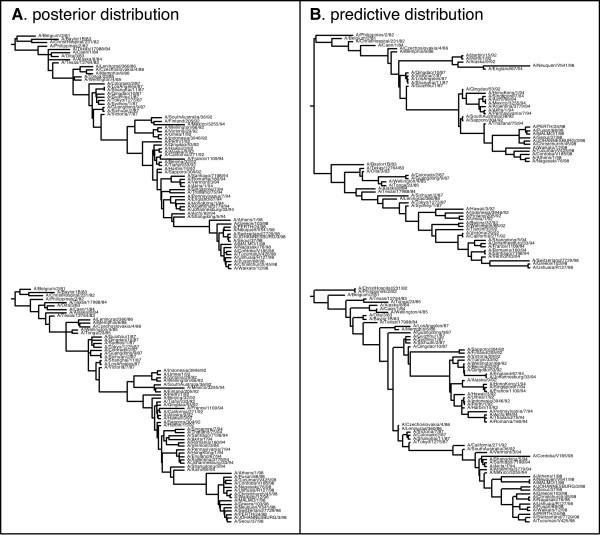
**Posterior and posterior predictive genealogies of human influenza A virus**. (A) A sample of two trees from the posterior distribution of the human influenza A virus data set. (B) The two matching trees simulated for the predictive distribution of the human influenza A virus data set. Obvious differences between the posterior and predictive trees are the shorter tree length and absence of deep splits in the posterior trees.

To demonstrate the ability of this method to detect non-neutrality, two additional data sets were analyzed. The first was a previously published data set of the E gene of the dengue-4 virus (*n *= 69, *L *= 1485) from Puerto Rico [42] spanning 17 years. The second was a data set of hemagglutinin sequences from human influenza A virus selected to have a similar time frame (1981–1998). These two viral data sets are both expected to exhibit the effects of adaptive selection, particularly influenza A virus, given the nature of their life histories [42-44]. As for the previous data sets, posterior and posterior predictive values were calculated for each of the summary statistics in Table 1. Under constant population size, the multivariate posterior predictive *p*-value = 0.0269 for the dengue-4 virus data set and = 0.0240 for the influenza A virus data set. Both of these data sets exhibited significantly more negative *D*_*F *_then expected under neutrality, suggesting that the relative length of the terminal branches is larger than expected in both data sets. Additionally, the human influenza A data set also had marginally more tree-imbalance than expected under neutrality, and for the dengue-4 data set, the age of the most recent common ancestor was significantly smaller than expected under neutrality. Figure 1 shows the posterior and predictive distributions for *D*_*F *_and tree length *T *for all four data sets. Figure 2 shows (A) two human influenza A virus trees from the posterior distribution of the exponential growth analysis along with (B) the corresponding simulated trees in the predictive distribution. These observations provide qualitative evidence for the ability to detect non-neutral evolutionary dynamics from tree shape as suggested in a recent review of the nascent field of phylodynamics [16].

#### Distinguishing selection from exponential growth

A criticism often leveled at tests of neutrality such as *D*_*F*_, is that significantly negative values of *D*_*F *_could signify exponential growth rather than non-neutral evolution. As demonstrated above the methodology employed here allows the demographic history to be described parametrically as part of the model. Therefore, inference and testing can both be achieved under a model of exponential growth. In this case, any additional departure from expectations cannot be attributed to exponential growth as the demographic signal is incorporated into the test via the predictive distribution. The results of the tests including exponential growth are also presented in Table 3. Interestingly, model selection by Bayes factors can not strongly reject constant populations in all of the data sets except for dengue-4. In the case of dengue-4, the log Bayes factor in favor of the exponential population model is approximately 8. However, the multivariate *p*-value for dengue-4 is no longer significant once exponential growth is incorporated. We can therefore distinguish between selection and growth in the dengue-4 and influenza data sets. In dengue-4, the departure from neutral expectations can be explained by an incorrect choice of demographic functions. Whereas in influenza, significant departures from neutral expectations are observed under both demographic scenarios. In contrast, there is little evidence of non-neutrality in the bear and HRSV data sets.

#### Simulations

For infinitely long sequences, for which no uncertainty in the underlying genealogy exists, *p*_*B *_behaves like a classical *p*-value. In the infinite data situation, the posterior distribution of **T**(·) collapses to a single point and equation (5) then returns the probability of observing a test statistic under the null hypothesis of selective neutrality as extreme as the test statistic of the data. In finite data situations, *p*_*B *_is stochastically less variable than a uniform distribution but with the same mean. This implies that the distribution of *p*_*B *_is more centered about 1/2 than a uniform random variable, leading to slightly more conservative tests when one choses small Type I error rates. To test the assertion that *p*_*B *_can still be interpreted as a *p*-value even when sequences are of short length and there is significant uncertainty in the underlying genealogy, a simulation study was undertaken. A number of replicate data sets (*n *= 100) were simulated and analyzed as follows:

1. A time-structured coalescent tree was simulated with sample times at 0, 300, 600, and 900 days, with 10 sequences at each time and a constant population-size parameter *N*_*e*_*τ *= 1500 (the product of *N*_*e *_and generation length in days).

2. DNA sequences of length 400 were simulated down the coalescent tree under an HKY85 + Γ model of substitution with parameters *κ *= 8, *σ *= 0.1, and *μ *= 4.0 × 10^-5 ^per site per day. Insertions and deletions were not simulated.

3. A Bayesian MCMC analysis was run on the resulting DNA sequence alignments using BEAST (Drummond and Rambaut 2004), assuming a constant population and an HKY85 + Γ model of substitution. The demographic and substitution parameters were all estimated assuming flat priors with conservative upper bounds.

4. For each state *p *of the MCMC, the sampled population size parameter *θ*^(*p*) ^~ *P*(⋯|**Y**) was used to generate a time-structure coalescent tree *G*^rep,(*p*)^. The set of trees for *p *= 1, ..., *P *is the predictive distribution of genealogies.

5. Using equation (5), the posterior distribution of genealogies was compared with the predictive distribution of genealogies, resulting in a *p*_*B *_value (using the *D*_*F *_statistic to summary the genealogies, as *D*_*F *_proved most powerful on the real data sets).

**Figure 3:**
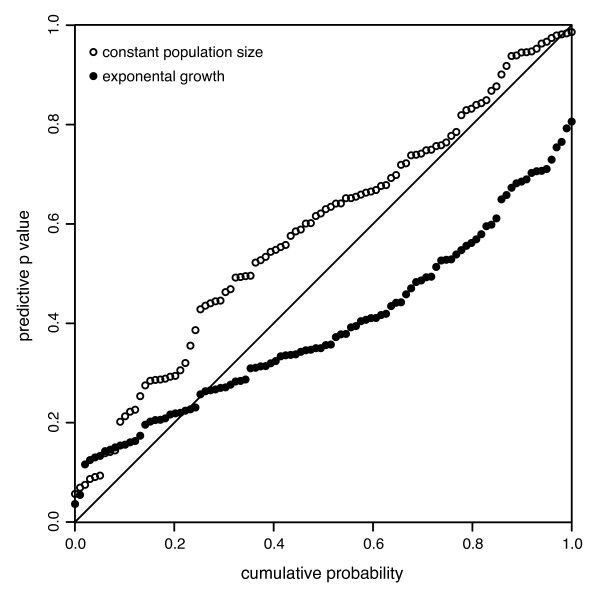
**Cumulative distribution of *p*_*B*_**. (Cumulative distribution of *p*_*B *_values (based on *D*_*F *_statistic) on 100 simulated data sets under a constant population (open circles) and an exponentially growing population (closed circles). The ideal behaviour for *p*_*B *_when applied to data simulated from the null distribution would be a uniform distribution (see main text for details). This plot shows that if the true demographic history is a constant population, then *p*_*B *_will be a good test of neutrality. However, if the true demographic history is exponential growth *p*_*B *_will be a conservative test, as can be seen by the lack of high *p*_*B *_values in the closed circles.

In the above scheme, the model used to simulate the data is the same as the model that we are testing against. Therefore we would expect the *p*_*B *_values to be distributed approximately uniformly between 0 and 1 under the null hypothesis. Figure 3 shows the cumulative probability distribution for the *p*_*B *_statistics calculated using the above scheme as well as for a 100 replicates where exponential growth (*N*_*e*_*τ *= 5000, *r *= 2 × 10^-3^) was assumed instead of a constant population size. For the case of constant population size, the *p*_*B *_values are distributed approximately uniformly, with 8 false positives (i.e. *p*_*B *_< 0.05) suggesting that the test is neither overly sensitive nor too conservative (Figure 3). However for the case of exponential growth the values of *p*_*B *_do not appear uniform with too few extreme values suggesting that the test would be conservative. These results strengthen the conclusion of a relative abundance of external-branch mutations in the viral data sets analyzed in this paper, because the significant statistics observed for real data sets both under constant-size and exponential growth assumptions would not be expected under neutrality.

## Conclusion

The results presented here demonstrate the utility of posterior predictive simulation for testing the goodness-of-fit of population genetic models of molecular evolution. In particular we tested the assumption of neutrality under both constant population size and exponential growth on four example data sets where temporally spaced data was available. In both dengue-4 and the human influenza A viruses there was a significant excess of mutations on terminal branches whether or not exponential growth was assumed. In contrast gene trees of HRSV and the d-loop of brown bears did not exhibit any significant departure from neutral expectations in terms of tree shape or genealogical distribution of mutations, although all four data sets had greater than average numbers of mutations on terminal branches relative to internal branches when compared to neutral expectations. Furthermore all four data sets had below average age of the root and below average tree length. In terms of tree-imbalance, both above and below average imbalances were observed for all three tree-imbalance statistics measured (*B*_1_, *C*_*n*_, *I*_*c*_).

This paper has been primarily concerned with demonstrating the utility of using existing summary statistics for testing neutrality in temporally spaced data sets. While we have demonstrated that existing statistics, such as *D*_*F *_can be successfully used to uncover non-neutral evolution it remains likely that better summary statistics may exist. We have described a method for comparing measures of tree shape with their expectations even if the tree shape statistic cannot be directly calculated from the sequence data. We hope that further development of test statistics of tree shape explicitly designed for temporally spaced data will proceed. By doing this we hope tests of recent phylodynamic theories [16] of genetic diversity and evolution in viral pathogens can be constructed. With the posterior predictive framework outlined here, new statistics should greatly increase our ability to detect non-neutral evolution and other departures from standard models of molecular evolution and population genetics. One potentially fruitful direction lies in examining violations of neutrality in the underlying substitution process, as well as in tree-shape. Efficient methods to detect substitution model violations by comparing the expected numbers of different classes of nucleotide substitutions have already been introduced [45]. This allows future work to combine appropriate summary statistics across the full model parameter space in order to maximize statistical power to detect non-neutral evolution.

Our reliance on posterior predictive simulation may raise the concern that the observed data **Y **for each example is "used" twice, first in generating the posterior distribution of model parameters and then in estimating the test-statistic employed to reject the null hypothesis. An alternative approach utilizing prior predictive simulation exists [46] and satisfies the above criticism. However, prior predictive simulation is undefined under improper prior distributions [47] and may not offer sufficient statistical power when vague priors are employed [48,49], such is the case in this work. In general, if the model parameters *G*, **Ω **and *θ *are well-estimated given **Y**, then the posterior predictive *p*-value yields results similar to classical *p*-values (when available), while the prior predictive assessment is highly sensitive to prior distribution choice [47]. In addition to these fully Bayesian predictive methods, Bayes factors [50] are effective model selection tools in phylogenetics [51]. A Bayes factor measures the relative likelihood of two competing models. To compute the Bayes factor in favor of the null model of neutrality, one must specify an alternative model. Unless the researcher has firm *a priori *knowledge about how neutrality might be violated in their data, we recommend starting with rejecting the null through these predictive methods and only then attempting the difficult task of non-neutral model construction and fitting.

Although both dengue-4 and the human influenza A viruses exhibit very ladder-like trees that are highly imbalanced, our analysis suggests that this amount of imbalance is not much more than would be expected given the sampling scheme and estimated effective population sizes. However it can be argued that small effective population sizes, by themselves, are evidence for selection. This is because effective population size (*N*_*e*_) is a measure of the number of productively replicating individuals, only when the population is evolving under conditions of neutrality. In the absence of any such prior assumptions, *N*_*e *_should be considered only as a surrogate measure of diversity in the population. Because diversity is reduced by selection, a low estimated *N*_*e *_could be a sign that the population process is being driven by natural selection. Nevertheless, the results presented here emphasize that ladder-like trees, by themselves, do not necessarily suggest selection. Consequently, interpretation of tree shape imbalance should not be made in the absence of an understanding of the expectations under the null model. Overall, for the example data sets chosen in this study, tree shape did not seem to be a powerful indicator of non-neutral evolution. Finally, by incorporating a demographic model into the test framework, we have ruled out exponential growth as the reason for significant predictive probabilities (*p*_*B*_) in all data sets besides dengue-4. Nevertheless there remain a number of alternative explanations for neutrality being rejected.

Both human influenza A and dengue-4 viruses show a significant excess of mutations on terminal branches when compared to the predictions of the best fitting parameters of the neutral model. These departures from neutrality lend insight into the process of molecular evolution in RNA viruses, and suggest that new models that take into account these departures need to be developed to accurately model their genetic variation. In contrast, at least with respect to tree shape and genealogical distribution of mutations, neutrality seems to be an approximately adequate model for the G gene of HRSV and the d-loop of brown bears. We hope that further application of posterior predictive simulation will shed light on the pattern of within-population genetic variation in a wide range of species and genetic elements.

## Methods

To assess selective neutrality in evolution, traditional test statistics summarize either the observed sequence data **Y **directly or the shape and inter-node distribution of a fixed gene genealogy *G *relating the sequences, where *G *is assumed known. In general, however, *G *is unknown *a priori *and must also be inferred from the sequence data with considerable uncertainty for measurably evolving populations [23]. This presents a difficulty for classical statistical tests. We overcome this short-fall in a Bayesian framework using posterior predictive assessment of model fit [33,47]. In this framework, we estimate *G *and its associated uncertainty from **Y **using a statistical model of molecular evolution and population demography and simultaneously compare a summary statistic of the random genealogy *G *to the statistic's expectation under neutrality. Our approach relies on assuming a statistical model for molecular evolution under neutrality. We employ a standard choice based on a continuous-time Markov chain process for nucleotide substitution [52] and an underlying coalescent process to generate the genealogy [53]. In particular, we assume the [37] (HKY85) substitution model with discrete – distributed rate heterogeneity across sites [38] parameterized by **Ω **= (*μ*, *κ*, *σ*). Parameter *μ *is the overall rate of mutation, *κ *is the transition/transversion bias and *σ *is the Gamma shape parameter. We assume a demographic coalescent process that allows for exponential population growth parameterized by *θ *= (*N*_*e*_*τ*, *r*). Parameter *N*_*e*_*τ *is the product of the effective population size and generation time and *r *is the exponential growth rate. Restricting *r *= 0 results in a constant population-size model. After assuming a prior distribution over (**Ω**, *θ*), we can approximate the posterior distribution

(1) *P*(*G*, **Ω**, *θ*|**Y**)

using Markov chain Monte Carlo (MCMC) techniques [54,55]. We refer interested readers to [22] for further details on prior choices and our MCMC approach. Simulation of (1) is readily available using the software BEAST [36].

With the tools to infer the random genealogy *G *and model parameters given sequence data in hand, we now consider summary statistics to assess the neutral model fit. Consider a vector of test statistics **T**(*G*) = [*T*_1_(*G*), ..., *T*_*K*_(*G*)] that summarize the shape of the genealogy *G*. Each element *T*_*k*_(*G*) for *k *= 1, ..., *K *serves as a unique mapping between *G *and the real numbers and generally returns a small value if *G *were generated by a neutral process and a large value otherwise. One such example for *T*_*k*_(*G*) is *D*_*F*_. Different *T*_*k*_(*G*) serve to detect different types of departures from the neutral tree form.

It is important to note that **T**(*G*) depends on an unknown model parameter in contrast to a classical test statistic that depends only on fixed quantities, such as the observed data **Y **or a fixed estimate of the genealogy $\hat{G}$. In the Bayesian literature, test statistics that depend on unknown model parameters (and also sometimes the data directly) are generally referred to as "discrepancy values" [48] to help differentiate them from classical measures. To simplify notation, we continue to refer to **T**(*G*) as a summary statistic with the implicit understanding that it is random and not directly observable. The advantage afforded by leaving **T**(*G*) a random variable is that we are now able to compare the discrepancy between the observed data **Y **and the posited neutral model as a whole, instead of between the data and the best fit of the model. To use **T**(*G*) to assess the model fit of neutrality, we consider the following thought experiment. Suppose we randomly simulate under a neutral model a genealogy *G*^rep ^from a replicated population almost identical to the population yielding the sequence data **Y**, where both populations share the same unknown demographic parameters *θ*, number of tips and tip-dates. Then, we compare quantities **T**(*G*^rep^) and **T**(*G*) given **Y**. Disparate values signify model misspecification caused by non-neutral evolutionary forces.

We recall that **T**(*G*^rep^) and **T**(*G*) given **Y **are not fixed values, but are random variables represented by probability distributions. As a consequence, we must integrate over all possible realizations weighed by their posterior probabilities to generate a test based on **T**(·). This process is called posterior predictive simulation [46-48,56]. Model selection and critique using posterior predictive simulation has had a successful history in phylogenetics [33,49,57-59].

The central distribution that we require is the posterior predictive distribution of the test statistic

(2) $$\begin{array}{l} P[T(G^{\text{rep}})|Y|=\\ \int_{G}\int_{\Omega }\int_{\theta }P[T(G^{\text{rep}})|\theta ]P(G,\Omega ,\theta |Y)\text{d}\theta \text{d}\Omega \text{d}G. \end{array}$$

In practice, one approximates the predictive distribution in (2) by first generating a posterior sample {*G*^(*p*)^, **Ω**^(*p*)^, *θ*^(*p*)^} for *p *= 1, ..., *P *from *P*(*G*, **Ω**, *θ*|**Y**). Then, for each *p*, one draws

(3) *G*^rep,(*p*) ^~ *P*(·|*θ*^(*p*)^),

where *P*(*G*^rep^|*θ*) describes a selectively neutral coalescent process. Finally, one tabulates **T**(*G*^rep,(*p*)^). We interpret this predictive distribution as a description of the values that **T**(·) generates when applied to genealogies from selectively neutral populations. To assess neutrality in the observed data, we compare the predictive distribution to the posterior distribution of the test statistic

(4) $$P[T(G)|Y]=\int_{\Omega }\int_{\theta }P[T(G),\Omega ,\theta |Y|\text{d}\Omega \text{d}\theta ,$$

approximated by tabulating **T**(*G*^(*p*)^) for *p *= 1, ..., *P*.

When the test statistic **T**(·) is univariate [33], assessing differences between predictive and posterior distributions can be done in two ways [47]. The first method is graphical, generating a scatterplot of {[**T**(*G*^rep,(*p*)^, **T**(*G*^(*p*)^)], *p *= 1, ..., *P*}. The second method is more formal, employing tail-area probabilities.

Let the posterior predictive *p*-value [48]

(5) *p*_*B *_= *P*[**T**(*G*^rep^) ≥ **T**(*G*)|**Y**],

then *p*_*B *_remains well-defined even though **T**(*G*^rep^) and **T**(*G*) given **Y **are not directly observable [47]. Probability *p*_*B *_shares many characteristics with a classical *p*-value; for example, *p*_*B *_can be viewed as its posterior mean and, under the null hypothesis of neutrality, *p*_*B *_is approximately distributed as a Uniform [0, 1) random variable [48]. Given these properties, we reject the selectively neutral model for extreme values of *p*_*B*_, say *p*_*B *_<*α *= 0.05 for strictly non-negative **T**(·) or *p*_*B *_<*α*/2 or *p*_*B *_> 1 - *α*/2 otherwise.

To calculate *p*_*B*_, a consistent estimator is

(6) $${\hat{p}}_{B}=\sum_{p=1}^{P}1\{T(G^{\text{rep},(p)})\geq T(G^{\left(p\right)})\}$$

where 1{·} is the indicator function, returning 1 if its argument is true and 0 otherwise.

When the test statistic **T**(·) is multivariate, we are able to detect a greater variety of departures from selective neutrality simultaneously, but a single tail-area probability becomes more troublesome to calculate. In this situation, we first standardize individual elements *T*_*k*_(·) such that var [*T*_*k*_(*G*)|**Y**] = 1 for all *k*. This places all measures on a common scale. We then generate scatterplots of the multivariate distributions. We agree with [47] in that comparing the posterior and predictive distributions graphically can provide more information than reporting a single *p*-value. For example, we can identify which components *T*_*k*_(·) in **T**(·) contribute greatest to the discrepancy between the data and a selectively neutral model.

To calculate a tail-area probability in the multivariate setting, we turn to the (squared) Mahalanobis distance in constructing a posterior predictive test [60]. Let $\hat{m}$ be an estimate of the predictive mean of **T**(*G*^rep^) and $\hat{V}$ be an estimate of its variance-covariance matrix, such that

(7) $$\begin{array}{lll} \hat{m} & = & \begin{array}{cc} \frac{1}{P}\sum_{p=1}^{P}T(G^{\text{rep},(p)}), & \text{and} \end{array}\\ \hat{V} & = & \frac{1}{P-1}\sum_{p=1}^{P}{\left[T(G^{\text{rep}(p)})-\hat{m}\right]}^{t}\left[T(G^{\text{rep},(p)})-\hat{m}\right], \end{array}$$

for *p *= 1, ..., *P*. Then, we define the (squared) Mahalanobis distance

(8) $$M(x)={\left(x-\hat{m}\right)}^{t}{\hat{V}}^{-1}(x-\hat{m}),$$

where we substitute **T**(*G*) for **x **when considering the distance's posterior distribution and **T**(*G*^rep^) for **x **when considering its predictive distribution. Mahalanobis distances are commonly used in discrimination analysis and classification. The metric of the Mahalanobis distance *M*(·) is the inverse of the variance-covariance matrix $\hat{V}$ of the predictive distribution and, as such, returns distances normalized relative to the multidimensional spread of the data under selective neutrality. Following in the light of Equation (5), we define the multivariate posterior predictive *p*-value

(9) *p*_*B *_= *P*[*M*(*G*^rep^) ≥ *M*(*G*)|**Y**].

A consistent estimator of the multivariate *p*_*B *_is readily available in the vain of Equation (6).

When it is unclear *a priori *which elements *T*_*k*_(·) provide the most power to reject selective neutrality, the multivariate approach side-steps the multiple testing problem inherent in examining each element independently. In these situations, we consider first using (9) as a global test with a fixed Type I Error rate *α *and then sub-selecting a small number of individual *T*_*k*_(·) for further univariate analysis. For researchers who begin by examining the *K *univariate analyses separately, we recommend applying a Bonferroni correction by decreasing the critical value cut-off from *α *to *α*/*K *per test. For large *K*, a Bonferrioni correction is overly conservative, especially when considering the potentially high correlation between *T*_*k*_(·). At this point, monitoring the false discovery rate [61] becomes more practical.

## Authors' contributions

AJD conceived the original idea and performed the initial data analysis and wrote the first draft of the paper. MAS constructed and performed the multivariate tests including re-creation of Figures 1 and 3 and Table 3. Both authors contributed to the final text.
